# Blockade of Alternative Complement Pathway in Dense Deposit Disease

**DOI:** 10.1155/2014/201568

**Published:** 2014-02-06

**Authors:** Aurore Berthe-Aucejo, Mathieu Sacquépée, Marc Fila, Michel Peuchmaur, Emilia Perrier-Cornet, Véronique Frémeaux-Bacchi, Georges Deschênes

**Affiliations:** ^1^Service de Pharmacie, Hôpital Robert Debré, 48 boulevard Sérurier, 75019 Paris, France; ^2^Service de Néphrologie, Centre Hospitalier Territorial de Nouvelle Calédonie, Gaston Bourret, BP J5, 98849 Nouméa, New Caledonia; ^3^Service de Néphrologie Pédiatrique, Hôpital Robert Debré, 48 boulevard Sérurier, 75019 Paris, France; ^4^Laboratoire d'Anatomopathologie, Hôpital Robert Debré, 48 boulevard Sérurier, 75019 Paris, France; ^5^Laboratoire d'Immunologie, Hôpital Européen Georges-Pompidou, 20-40 rue Leblanc, 75015 Paris, France

## Abstract

A patient aged 17 with dense deposit disease associated with complement activation, circulating C3 Nef, and Factor H mutation presented with nephrotic syndrome and hypertension. Steroid therapy, plasma exchange, and rituximab failed to improve proteinuria and hypertension despite a normalization of the circulating sC5b9 complex. Eculizumab, a monoclonal antibody directed against C5, was used to block the terminal product of the complement cascade. The dose was adapted to achieve a CH50 below 10%, but proteinuria and blood pressure were not improved after 3 months of treatment.

## 1. Introduction

Dense deposit disease or DDD (formerly referred to as membranoproliferative glomerulonephritis type II) is a rare disease affecting less than 2 people per million, both adults and children. Nephrotic syndrome, severe hypertension, and progression to chronic renal failure are usually observed in patients with DDD. The histological pattern in light microcopy is limited to an enlargement of the mesangium with a mild mesangial cell hypercellularity [1]. Electron microscopy allows us to evidence electron-dense enlargement of the glomerular basement membrane that specially affects the lamina densa. Paradoxically, the precise composition of the dense deposits is not really known [2], while C3 fraction is only seen in the margin of the dense deposits but not within the dense deposits [1]. Complement activation with low C3 levels due to complement alternative pathway dysregulation is mostly due to the presence of a circulation C3 nephritic factor (C3 NeF) which is an autoantibody that stabilizes C3 convertase. In addition, mutations in the factor H gene have also been reported in a few patients [3]. Steroid therapy can be used but the efficacy was not proven. Specific treatment can be proposed like plasma exchanges. They is used to clear the C3 NeF and restore a normal complement balance. They have also shown encouraging results. New therapeutic approaches such as rituximab (anti-CD20) or eculizumab (anti-C5) could be proposed [1]. Eculizumab, a monoclonal antibody directed against complement C5 that blocks the final products of complement activation, might subsequently be considered as a relevant treatment in DDD. Here, we present the case of a patient presenting with DDD, in whom eculizumab was tried during 3 months.

## 2. Case Presentation

**Figure 1:**
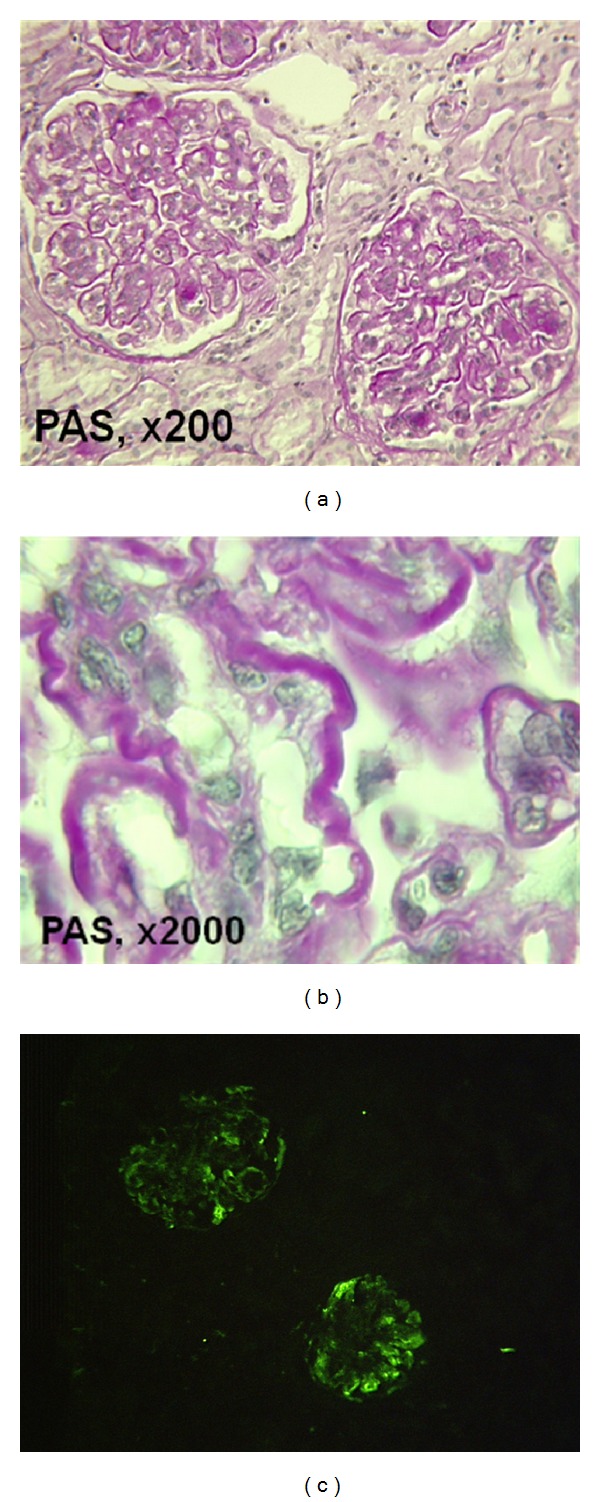
(a) Type II membranoproliferative glomerulonephritis characterized by mesangial matrix and cellular increases is responsible for a lobular accentuation associated with a diffuse and intense staining of the peripheral basement membrane (periodic acid-Schiff [PAS], magnification [G]: ×200). (b) The diffuse and intense staining of the peripheral basement membrane indicates the presence of dense deposit material (PAS, G ×2000). (c) Immunofluorescence techniques show segmental pseudo linear and granular IgM deposits along the peripheral capillary wall (fluorescein isothiocyanate anti-IgM, G ×100).

**Figure 2:**
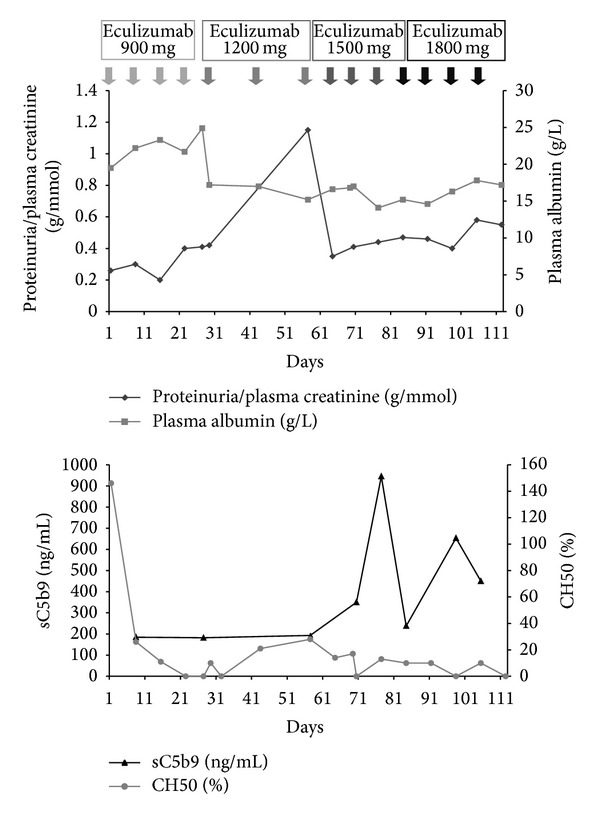
Response to eculizumab therapy in dense deposit disease.

The patient was a young man aged 17 and born from unrelated parents. In September 2007, at the age of 15, hypertension and nephrotic syndrome (proteinuria = 2.14 g/24 h, plasma albumin = 25 g/L) led to performing a renal biopsy and he was diagnosed with a DDD in October 2007. Hepatitis C virus (HCV) and human immunodeficiency virus (HIV) tests were negative and the patient was vaccinated against hepatitis B virus (HBV). Low C3 (538 mg/L; normal value 660–1250) with normal C4 (160 mg/L; normal values 93–380) levels were evidenced and related to a circulating C3 NeF. Plasma level of antigenic factor H was 54% (normal value 65–140), while those of factor B and factor I were normal. In addition, gene sequencing analysis of complement factor H gene showed an heterozygous mutation (p. R232X) located in SCR 4 (short consensus repeats) leading to a deficiency in factor H. Renal function was within normal limits at this period (serum creatinine = 59 *μ*mol/L). The patient received 10 plasma exchanges against fresh frozen plasma from April 2008 to June 2008 and then four injections of rituximab, a monoclonal antibody directed against CD20, that led to B cell depletion during several months and was supposed to control the C3 NeF. This treatment showed no efficacy on nephrotic syndrome. The plasma level of sC5b9 complex level was high at 755 ng/mL in February 2008 and was normalized (<600 ng/mL) before plasma exchange. Oral alternative-day steroid therapy (prednisolone 40 mg/48 h) as well as ramipril and irbesartan was given from June 2008, but the patient disrupted the medical follow-up during 2 years. In March 2010, the blood pressure was 132/85, proteinuria was 2.55 g/L (0.26 g/mmol of creatinine), serum albumin was 16.7 g/L, and eGFR was 93 mL/min/1.73/m² according to the 2009 Schwartz formula. A second renal biopsy was realized and showed dense deposits in 100% of glomerulus and 15% of interstitial fibrosis (Figures 1(a), 1(b), and 1(c)). The patient was vaccinated with Menactra to prevent *Neisseria meningitidis* infection as a preparation prior to introduce eculizumab. Initial schedule was similar to those proposed in patients with atypical haemolytic uremic syndrome, namely, 900 mg every week for 4 weeks and then 1200 mg on week 5 and every 2 weeks. Treatment was associated with penicillin V treatment. After 5 weeks of treatment, CH50 level decreased from 146% to an undetectable plasma level. From week 8, CH50 resumed to be 25% and the dose of eculizumab was subsequently increased to 1500 mg every week and by day 84 to 1800 mg every week in order to achieve a complete and continuous blockade of CH50 (below 10%, Figure 2). At the time of the first injection, the plasma level of sC5b9 was normal and remained in the normal range during the first 7 injections. Paradoxically, the level rose up to 945 ng/mL, while the dose of eculizumab was increased to 1500 mg per week and more. Renal function, blood pressure, and proteinuria were unchanged after 3 months of treatment (Figure 2). Plasma albumin transiently increased to 25 g/L by day 25 and then after return to the initial level or below (Figure 1). Subsequently, eculizumab was stopped after 14 injections and 3.5 months of continuous treatment. No side effect was observed during the treatment.

The patient is lost to follow-up for one year. At last control, nephrotic syndrome was persistent and the patient was considered to have a normal renal function.

## 3. Discussion

The diagnosis of DDD is frequently done in children below 15 years of age, with half of them progressing to end-stage renal failure in less than 10 years. In immunofluorescence, C3 deposits feature a “railroad track” on both sides of dense deposits, while IgG deposits are lacking. In most patients, a positive C3 NeF, an autoantibody that stabilizes the C3 convertase, is associated with an alternative pathway C3 consumption. Therefore, the fluid phase-restricted complement alternative pathway dysregulation with a continuously activated and consumed C3 should be a prerequisite for the development of DDD [4]. Most treatments of DDD are based on results obtained in short case series due to the rarity of the disease [5]. Steroids are usually considered as ineffective in DDD although several pediatric case reports showed improvement of proteinuria. Plasma replacement therapy has also been reported in single case reports and might stabilize the creatinine clearance in the rapidly progressive forms of the disease. According to the permanent activation of the complement alternative pathway, the treatment of DDD with eculizumab could be a relevant alternative treatment and has been reported to reduce the level of proteinuria in the first case reports [6–9]. Nevertheless, the most recent series of 6 patients treated with eculizumab, whose 3 patients were diagnosed with DDD, showed variability of responses from no effect to a partial or a transient effect [7]. Consistently, our patient did not show any benefit of this very expensive drug, despite the close control of a complete blockade of the final complement byproducts during 3.5 months. Among the many causes of failure, the duration of the disease over 2 years prior to the treatment and a period of active therapy limited to 3 months are the main limits in the interpretation of this case report. Moreover, discordance between sC5b9 and CH50 levels was observed and not explained. Indeed, sC5b9 level paradoxically rose after 10 injections of eculizumab, while CH50 level was undetectable suggesting a complete blockade of the alternative pathway. Failure can be explained by a normal level of sC5b9 at the time of the first injection of eculizumab, while previous reports showing an efficacy of eculizumab include patients with an initial high plasma level of sC5b9 complex [7, 9].

We conclude that the short-term blockade of complement is not systematically successful in all patients with DDD. As previously suggested, additional research is needed to isolate the subgroup of patients, in whom eculizumab could be used with success and will certainly improve our understanding of the disease.

## References

[1] Smith RJH, Alexander J, Barlow PN (2007). New approaches to the treatment of dense deposit disease. *Journal of the American Society of Nephrology*.

[2] Sethi S, Gamez JD, Vrana JA (2009). Glomeruli of Dense Deposit Disease contain components of the alternative and terminal complement pathway. *Kidney International*.

[3] Servais A, Noël L-H, Roumenina LT (2012). Acquired and genetic complement abnormalities play a critical role in dense deposit disease and other C3 glomerulopathies. *Kidney International*.

[4] Martínez-Barricarte R, Heurich M, Valdes-Cañedo F (2010). Human C3 mutation reveals a mechanism of dense deposit disease pathogenesis and provides insights into complement activation and regulation. *Journal of Clinical Investigation*.

[5] Appel GB, Cook HT, Hageman G (2005). Membranoproliferative glomerulonephritis type II (dense deposit disease): an update. *Journal of the American Society of Nephrology*.

[6] Vivarelli M, Pasini A, Emma F (2012). Eculizumab for the treatment of dense-deposit disease. *New England Journal of Medicine*.

[7] Bomback AS, Smith RJ, Barile GR (2012). Eculizumab for dense deposit disease and C3 glomerulonephritis. *Clinical Journal of the American Society of Nephrology*.

[8] Daina E, Noris M, Remuzzi G (2012). Eculizumab in a patient with dense-deposit disease. *New England Journal of Medicine*.

[9] Radhakrishnan S, Lunn A, Kirschfink M (2012). Eculizumab and refractory membranoproliferative glomerulonephritis. *New England Journal of Medicine*.

